# Subject Index Volume 9

**DOI:** 10.1111/jdi.12924

**Published:** 2018-11-04

1p13

Association between 1p13 polymorphisms and peripheral arterial disease in a Chinese population with diabetes, Qin 1189–1195

α‐cell mass

Human pancreatic α‐ to β‐cell area ratio increases after type 2 diabetes onset, Fujita 1270–1282

α‐cell proliferation

Human pancreatic α‐ to β‐cell area ratio increases after type 2 diabetes onset, Fujita 1270–1282

α‐glucosidase inhibitor

Efficacy and safety of combination therapy with an α‐glucosidase inhibitor and a dipeptidyl peptidase‐4 inhibitor in patients with type 2 diabetes mellitus: A systematic review with meta‐analysis, Min 893–902

accuracy

Performance of a new real‐time continuous glucose monitoring system: A multicenter pilot study, Zhou 286–293

acromegaly

Marked alteration of glycemic profile surrounding lanreotide administration in acromegaly: A case report, Tanaka 223–225

acute‐onset type 1 diabetes

Complete loss of insulin secretion capacity in type 1A diabetes patients during long‐term follow up, Uno 806–812

add‐on to liraglutide

Sodium‐glucose cotransporter‐2 inhibitor luseogliflozin added to glucagon‐like peptide 1 receptor agonist liraglutide improves glycemic control with bodyweight and fat mass reductions in Japanese patients with type 2 diabetes: A 52‐week, open‐label, single‐arm study, Seino 332–340

adiponectin

Serum adiponectin and insulin secretion: A direct or inverse association?, Nakamura 1106–1109

adipsin

Relationship between serum adipsin and the first phase of glucose‐stimulated insulin secretion in individuals with different glucose tolerance, Zhou 1128–1134

age

Age at natural menopause and risk of diabetes in adult women: Findings from the China Kadoorie Biobank study in the Zhejiang area, Wang 762–768

age at menarche

Impact of age at menarche on obesity and glycemic control in Japanese patients with type 2 diabetes: Fukuoka Diabetes Registry, Sumi 1216–1223

alpha‐glucosidase inhibitors

Meta‐analysis and critical review on the efficacy and safety of alpha‐glucosidase inhibitors in Asian and non‐Asian populations, Gao 321–331

amino acid catabolism

Regulation of amino acid metabolism and α‐cell proliferation by glucagon, Hayashi 464–472

amino acid transporter

Regulation of amino acid metabolism and α‐cell proliferation by glucagon, Hayashi 464–472

a new hip index

Assessing a new hip index as a risk predictor for diabetes mellitus, He 799–805

angiopoietin‐like protein 8

Angiopoietin‐like protein 8/betatrophin correlates with hepatocellular lipid content independent of insulin resistance in non‐alcoholic fatty liver disease patients, Hong 952–958

annual medical expenditure

Impact of obesity on annual medical expenditures and diabetes care in Japanese patients with type 2 diabetes mellitus, Kusunoki‐Tsuji 776–781

antihypertensive drugs

Current status of achieving blood pressure target and its clinical correlates in Japanese type 2 diabetes (JDDM45), Yokoyama 594–601

anti‐programmed death 1 antibody

Aggravation of diabetes, and incompletely deficient insulin secretion in a case with type 1 diabetes‐resistant human leukocyte antigen DRB1*15:02 treated with nivolumab, Matsumura 438–441

apolipoprotein B‐100

Comparison of effects of anagliptin and alogliptin on serum lipid profile in type 2 diabetes mellitus patients, Kurozumi 360–365

aristaless‐related homeobox

Human pancreatic α‐ to β‐cell area ratio increases after type 2 diabetes onset, Fujita 1270–1282

arterial stiffness

Grading effect of abnormal glucose status on arterial stiffness and a new threshold of 2‐h post‐load glucose based on a Chinese community study, Liu 616–622

Asia

Oral glucose lowering with linagliptin and metformin compared with linagliptin alone as initial treatment in Asian patients with newly diagnosed type 2 diabetes and marked hyperglycemia: Subgroup analysis of a randomized clinical trial, Ma 579–586
Cross‐sectional survey of biosimilar insulin utilization in Asia: The Joint Asia Diabetes Evaluation Program, Gani 1312–1322

Asian

Meta‐analysis and critical review on the efficacy and safety of alpha‐glucosidase inhibitors in Asian and non‐Asian populations, Gao 321–331
No disparity of the efficacy and all‐cause mortality between Asian and non‐Asian type 2 diabetes patients with sodium–glucose cotransporter 2 inhibitors treatment: A meta‐analysis, Cai 850–861

β‐cell

Insulin‐producing cells derived from ‘induced pluripotent stem cells’ of patients with fulminant type 1 diabetes: Vulnerability to cytokine insults and increased expression of apoptosis‐related genes, Hosokawa 481–493

β‐cell function

Plasma soluble leptin receptor levels are associated with pancreatic β‐cell dysfunction in patients with type 2 diabetes, Morioka 55–62

Retrospective analysis of liraglutide and basal insulin combination therapy in Japanese type 2 diabetes patients: The association between remaining β‐cell function and the achievement of the glycated hemoglobin target 1 year after initiation, Usui 822–830

bariatric surgery

Role of gut microbiota, bile acids and their cross‐talk in the effects of bariatric surgery on obesity and type 2 diabetes, Liu 13–20

basal insulin

Retrospective analysis of liraglutide and basal insulin combination therapy in Japanese type 2 diabetes patients: The association between remaining β‐cell function and the achievement of the glycated hemoglobin target 1 year after initiation, Usui 822–830

basal insulin peglispro

Open‐label, randomized study comparing basal insulin peglispro and insulin glargine, in combination with oral antihyperglycemic medications, in insulin‐naïve Asian patients with type 2 diabetes, Hirose 100–107

basal metabolism

Role of vitamin D in energy and bone metabolism in postmenopausal women with type 2 diabetes mellitus: A 6‐month follow‐up evaluation, Ogata 211–222

basal supported oral therapy

Efficacy and safety of switching from basal insulin to once‐daily insulin degludec/insulin aspart in Japanese patients with inadequately controlled type 2 diabetes: A 4‐week, randomized, open‐label, treat‐to‐target study, Nagai 567–572

battery

Safety of the batteries and power units used in insulin pumps: A pilot cross‐sectional study by the Association for the Study of Innovative Diabetes Treatment in Japan, Murata 903–907

biguanide

Effects of dosage and dosing frequency on the efficacy and safety of high‐dose metformin in Japanese patients with type 2 diabetes mellitus, Kanto, 587–593

bile acids

Role of gut microbiota, bile acids and their cross‐talk in the effects of bariatric surgery on obesity and type 2 diabetes, Liu 13–20

biological marker

Ficolin‐3/adiponectin ratio for the prediction of gestational diabetes mellitus in pregnant women, Yuan 403–410

biomarker

Metabolomics window into diabetic complications, Wu 244–255
Microribonucleic acid‐192 as a specific biomarker for the early diagnosis of diabetic kidney disease, Yang 602–609

biosimilar

Cross‐sectional survey of biosimilar insulin utilization in Asia: The Joint Asia Diabetes Evaluation Program, Gani 1312–1322

blood glucose

Grading effect of abnormal glucose status on arterial stiffness and a new threshold of 2‐h post‐load glucose based on a Chinese community study, Liu 616–622

blood glucose self‐monitoring

Factors associated with greater benefit of a national reimbursement policy for blood glucose test strips in adult patients with type 1 diabetes: A prospective cohort study, Jin 549–557

blood lipids

Prevalence, treatment patterns and control rates of metabolic syndrome in a Chinese diabetic population: China Cardiometabolic Registries 3B study, Jing 789–798

blood pressure

Blood pressure circadian rhythms and adverse outcomes in type 2 diabetes patients diagnosed with orthostatic hypotension, Chang 383–388

blood pressure control

Current status of achieving blood pressure target and its clinical correlates in Japanese type 2 diabetes (JDDM45), Yokoyama 594–601

body mass index

Impact of obesity on mortality in patients with diabetes: Meta‐analysis of 20 studies including 250,016 patients, Gao 44–54

none metabolism

Role of vitamin D in energy and bone metabolism in postmenopausal women with type 2 diabetes mellitus: A 6‐month follow‐up evaluation, Ogata 211–222

bullous pemphigoid

Bullous pemphigoid associated with dipeptidyl peptidase‐4 inhibitors: A report of five cases, Yoshiji 445–447

C‐peptide

Normal meal tolerance test is preferable to the glucagon stimulation test in patients with type 2 diabetes that are not in a hyperglycemic state: Comparison with the change of C‐peptide immunoreactivity, Fujioka 274–278

cardiovascular disease

Ratio of visceral‐to‐subcutaneous fat area predicts cardiovascular events in patients with type 2 diabetes, Fukuda 396–402

carotid atherosclerosis

Characteristics of carotid atherosclerosis in elderly patients with type 2 diabetes at different disease course, and the intervention by statins in very elderly patients, Chen 389–395

cell death‐inducing DFF45‐like effector C

Cell death‐inducing DFF45‐like effector C gene silencing alleviates pulmonary vascular remodeling in a type 2 diabetic rat model, Sui 741–752

cell therapy

iPSC technology‐based regenerative therapy for diabetes, Kondo 234–243

Charcot neuroarthropathy

Corneal confocal microscopy detects severe small fiber neuropathy in diabetic patients with Charcot neuroarthropathy, Khan 1167–1172

childhood obesity

Serum γ‐glutamyltransferase level and metabolic syndrome in children and adolescents: Korean National Health and Nutrition Examination Survey, Park 522–528

children

Glycemic extremes are related to cognitive dysfunction in children with type 1 diabetes: A meta‐analysis, He 1342–1353

Chinese

Preliminary screening of mutations in the glucokinase gene of Chinese patients with gestational diabetes, Wang 199–203

cholecalciferol

Association between serum 25‐hydroxyvitamin D and glycated hemoglobin levels in type 2 diabetes patients with chronic kidney disease, Lim 375–382

cholesterol transport

Dipeptidyl peptidase 4 inhibitor anagliptin ameliorates hypercholesterolemia in hypercholesterolemic mice through inhibition of intestinal cholesterol transport, Goto 1261–1269

chronic inflammation

Molecular mechanism of obesity‐induced ‘metabolic’ tissue remodeling, Tanaka 256–261

clinical

Difference in normal limit values of nerve conduction parameters between Westerners and Japanese people might need to be considered when diagnosing diabetic polyneuropathy using a Point‐of‐Care Sural Nerve Conduction Device (NC‐stat^®^/DPNCheck^™^), Hirayasu 1173–1181

cognitive function

Glycemic extremes are related to cognitive dysfunction in children with type 1 diabetes: A meta‐analysis, He 1342–1353

cohort studies

Relationship between social engagement and diabetes incidence in a middle‐aged population: Results from a longitudinal nationwide survey in Japan, Shibayama 1060–1066

cohort study

Real‐life glycemic control in patients with type 2 diabetes treated with insulin therapy: A prospective, longitudinal cohort study (Diabetes Distress and Care Registry at Tenri [DDCRT 9]), Fujita 294–302

Failure of monotherapy in clinical practice in patients with type 2 diabetes: The Korean National Diabetes Program, Jeon 1144–1152

Commentary

Incretin concept revised: The origin of the insulinotropic function of glucagon‐like peptide‐1 – the gut, the islets or both?, Yabe 21‐24
Regulation of glucagon‐like peptide‐1 sensitivity by gut microbiota dysbiosis, Yamane 262–264
Unfolding link between diabetes and cancer, Noto 473–474
Epigenetic dysregulation in pancreatic islets and pathogenesis of type 2 diabetes, Yokoi 475–477
Effect of real‐time continuous glucose monitoring on hypoglycemia in adult type 1 diabetes patients, Jia 478–480
Novel strategy for screening of diabetic retinopathy, Takagi 726–727
Cardiovascular safety of long‐acting insulin analogs in type 2 diabetes patients: Is there a better basal insulin?, Chang 728–730
Role of inflammatory biomarkers in diabetic peripheral neuropathy, Jin 1016–1018
Diabetes‐related cognitive dysfunction: Hyperglycemia in the early stage might be a key?, Umegaki 1019–1021
Impacts of tight multifactorial intervention in patients with type 2 diabetes: Implications from the Japan Diabetes Outcome Intervention Trial 3, Araki 1022–1024
How does glucagon‐like peptide 1 stimulate human β‐cell proliferation? A lesson from islet graft experiments, Fujitani 1255–1257
History of gestational diabetes and future cardiovascular disease: What have we learned?, Hsu 1258–1260

complication I – nerve

Difference in normal limit values of nerve conduction parameters between Westerners and Japanese people might need to be considered when diagnosing diabetic polyneuropathy using a Point‐of‐Care Sural Nerve Conduction Device (NC‐stat^®^/DPNCheck™), Hirayasu 1173–1181

congenital abnormalities

Insulin degludec in the first trimester of pregnancy: Report of two cases, Milluzzo 629–631

continuous glucose monitoring

Continuous glucose monitoring reveals hypoglycemia risk in elderly patients with type 2 diabetes mellitus, Ishikawa 69–74
Twenty‐four‐hour variations in blood glucose level in Japanese type 2 diabetes patients based on continuous glucose monitoring, Hajime 75–82
Comparison of morning basal + 1 bolus insulin therapy (insulin glulisine + insulin glargine 300 U/mL vs insulin lispro + insulin glargine biosimilar) using continuous glucose monitoring: A randomized crossover study, Takeishi 91–99
Performance of a new real‐time continuous glucose monitoring system: A multicenter pilot study, Zhou 286–293
Efficacy of ipragliflozin as monotherapy or as add‐on therapy with other oral antidiabetic medications for treating type 2 diabetes in Japanese patients with inadequate glycemic control: A subgroup analysis based on patient characteristics, Osonoi 341–353
Biphasic insulin aspart‐30 reduces glycemic variability to a greater degree than insulin detemir: A randomized controlled trial of once‐daily insulin regimens using continuous glucose monitoring, Tsujino 573–578

continuous positive airway pressure

Sleep apnea and type 2 diabetes, Muraki 991–997

continuous subcutaneous insulin infusion

Safety of the batteries and power units used in insulin pumps: A pilot cross‐sectional study by the Association for the Study of Innovative Diabetes Treatment in Japan, Murata 903–907

continuous venovenous hemodiafiltration

High‐volume continuous venovenous hemodiafiltration plus resin hemoperfusion improves severe metformin‐associated toxicity, Liu 975–978

corneal confocal microscopy

Corneal confocal microscopy detects severe small fiber neuropathy in diabetic patients with Charcot neuroarthropathy, Khan 1167–1172

corneal nerves

Corneal confocal microscopy detects severe small fiber neuropathy in diabetic patients with Charcot neuroarthropathy, Khan 1167–1172

coronary plaque

Comparison of the relationship between multiple parameters of glycemic variability and coronary plaque vulnerability assessed by virtual histology–intravascular ultrasound, Otowa‐Suematsu 610–615

cost‐effectiveness

Prevention of renal failure in Chinese patients with newly diagnosed type 2 diabetes: A cost‐effectiveness analysis, Wu 152–161

costs

Characteristics of frequent emergency department users with type 2 diabetes mellitus in Korea, Ustulin 430–437

cross‐reactivity

Antibodies against H1N1 influenza virus cross‐react with α‐cells of pancreatic islets, Qi 265–269

cultural psychology

Cross‐cultural comparison of predictors for self‐care behaviors in patients with type 2 diabetes, Ikeda 1212–1215

curcumin

Curcumin attenuates high glucose‐induced inflammatory injury through the reactive oxygen species–phosphoinositide 3‐kinase/protein kinase B–nuclear factor‐κB signaling pathway in rat thoracic aorta endothelial cells, Zhang 731–740

database analysis

Incidence rate and patient characteristics of severe hypoglycemia in treated type 2 diabetes mellitus patients in Japan: Retrospective Diagnosis Procedure Combination database analysis, Ikeda 925–936

diabetes

Impact of obesity on mortality in patients with diabetes: Meta‐analysis of 20 studies including 250,016 patients, Gao 44–54
Characteristics of frequent emergency department users with type 2 diabetes mellitus in Korea, Ustulin 430–437
Cell death‐inducing DFF45‐like effector C gene silencing alleviates pulmonary vascular remodeling in a type 2 diabetic rat model, Sui 741–752
Effects of atorvastatin on autophagy in skeletal muscles of diabetic rats, Yang 753–761
Age at natural menopause and risk of diabetes in adult women: Findings from the China Kadoorie Biobank study in the Zhejiang area, Wang 762–768
Assessment of resting energy expenditure and body composition in Japanese pregnant women with diabetes, Eto 959–966
Sleep apnea and type 2 diabetes, Muraki 991–997

diabetes mellitus

Marked alteration of glycemic profile surrounding lanreotide administration in acromegaly: A case report, Tanaka 223–225
Blood pressure circadian rhythms and adverse outcomes in type 2 diabetes patients diagnosed with orthostatic hypotension, Chang 383–388
Prevalence of lipohypertrophy in insulin‐treated diabetes patients: A systematic review and meta‐analysis, Deng 536–543
Insulin degludec in the first trimester of pregnancy: Report of two cases, Milluzzo 629–631
Prevalence, treatment patterns and control rates of metabolic syndrome in a Chinese diabetic population: China Cardiometabolic Registries 3B study, Jing 789–798
Assessing a new hip index as a risk predictor for diabetes mellitus, He 799–805
Sodium–glucose cotransporter 2 inhibitor, tofogliflozin, shows better improvements of blood glucose and insulin secretion in patients with high insulin levels at baseline, Tobe 862–869
Pro‐apoptotic effects of micro‐ribonucleic acid‐365 on retinal neurons by targeting insulin‐like growth factor‐1 in diabetic rats: An *in vivo* and *in vitro* study, Zheng 1041–1051

diabetes mellitus type 2

Oral glucose lowering with linagliptin and metformin compared with linagliptin alone as initial treatment in Asian patients with newly diagnosed type 2 diabetes and marked hyperglycemia: Subgroup analysis of a randomized clinical trial, Ma 579–586

diabetes self‐care

Cross‐cultural comparison of predictors for self‐care behaviors in patients with type 2 diabetes, Ikeda 1212–1215

diabetic complications

Metabolomics window into diabetic complications, Wu 244–255

diabetic kidney disease

Prevention of renal failure in Chinese patients with newly diagnosed type 2 diabetes: A cost‐effectiveness analysis, Wu 152–161
Microribonucleic acid‐192 as a specific biomarker for the early diagnosis of diabetic kidney disease, Yang 602–609

diabetic nephropathies

Association between serum 25‐hydroxyvitamin D and glycated hemoglobin levels in type 2 diabetes patients with chronic kidney disease, Lim 375–382

diabetic nephropathy

Subcutaneous injection of hydrogen gas is a novel effective treatment for type 2 diabetes, Zhang 83–90
Association between poor psychosocial conditions and diabetic nephropathy in Japanese type 2 diabetes patients: A cross‐sectional study, Ninomiya 162–172
Tryptophan as a surrogate prognostic marker for diabetic nephropathy, Chou 366–374

diabetic nephropathy

Clinical significance of serum interleukin‐8 and soluble tumor necrosis factor‐like weak inducer of apoptosis levels in patients with diabetic nephropathy, Liu 1182–1188

diabetic neuropathy

Diabetic polyneuropathy is a risk factor for decline of lower extremity strength in patients with type 2 diabetes, Nomura 186–192

diabetic peripheral arterial disease

Association between 1p13 polymorphisms and peripheral arterial disease in a Chinese population with diabetes, Qin 1189–1195

diabetic polyneuropathy

Diabetic neuropathy and the sensory neuron: New aspects of pathogenesis and their treatment implications, Kobayashi 1239–1254

diabetic retinopathy

Trends in diabetic retinopathy and related medical practices among type 2 diabetes patients: Results from the National Insurance Service Survey 2006–2013, Song 173–178
Predictors of postoperative bleeding after vitrectomy for vitreous hemorrhage in patients with diabetic retinopathy, Motoda 940–945

diagnosis and pathophysiology

Difference in normal limit values of nerve conduction parameters between Westerners and Japanese people might need to be considered when diagnosing diabetic polyneuropathy using a Point‐of‐Care Sural Nerve Conduction Device (NC‐stat^®^/DPNCheck^™^), Hirayasu 1173–1181

dietary fiber

Postprandial glucose‐lowering effect of premeal consumption of protein‐enriched, dietary fiber‐fortified bar in individuals with type 2 diabetes mellitus or normal glucose tolerance, Bae 1110–1118

diet‐induced obesity

Determination of peripheral neuropathy in high‐fat diet fed low‐dose streptozotocin‐treated female C57Bl/6J mice and Sprague–Dawley rats, Coppey 1033–1040

dietary behavior

Dietary intake habits and the prevalence of nocturia in Japanese patients with type 2 diabetes mellitus, Furukawa 279–285

dietary habits

Relationship between deterioration of glycated hemoglobin‐lowering effects in dipeptidyl peptidase‐4 inhibitor monotherapy and dietary habits: Retrospective analysis of Japanese individuals with type 2 diabetes, Kuwata 1153–1158

dipeptidyl peptidase 4 inhibitor

Comparison of effects of anagliptin and alogliptin on serum lipid profile in type 2 diabetes mellitus patients, Kurozumi 360–365
Addition of dipeptidyl peptidase‐4 inhibitors to insulin treatment in type 2 diabetes patients: A meta‐analysis, Yang 813–821
Efficacy and safety of combination therapy with an α‐glucosidase inhibitor and a dipeptidyl peptidase‐4 inhibitor in patients with type 2 diabetes mellitus: A systematic review with meta‐analysis, Min 893–902
Dipeptidyl peptidase 4 inhibitor anagliptin ameliorates hypercholesterolemia in hypercholesterolemic mice through inhibition of intestinal cholesterol transport, Goto 1261–1269

dipeptidyl peptidase‐4 inhibitors

Bullous pemphigoid associated with dipeptidyl peptidase‐4 inhibitors: A report of five cases, Yoshiji 445–447
Oral glucose lowering with linagliptin and metformin compared with linagliptin alone as initial treatment in Asian patients with newly diagnosed type 2 diabetes and marked hyperglycemia: Subgroup analysis of a randomized clinical trial, Ma 579–586
Relationship between deterioration of glycated hemoglobin‐lowering effects in dipeptidyl peptidase‐4 inhibitor monotherapy and dietary habits: Retrospective analysis of Japanese individuals with type 2 diabetes, Kuwata 1153–1158

disease model

iPSC technology‐based regenerative therapy for diabetes, Kondo 234–243

disposition index

Serum adiponectin and insulin secretion: A direct or inverse association?, Nakamura 1106–1109

Editorial

Diabetes related fatigue sarcopenia, frailty, Lien 3–4
Microencapsulation for cell therapy of type 1 diabetes mellitus: The interplay between common beliefs, prejudices and real progress, Calafiore 231–233
Molecular clock as a regulator of β‐cell function, Taguchi, 453–456
Mechanisms of metformin action: In and out of the gut, Minamii 701–703
Bariatric and metabolic surgery in Asia: Where are we, and where are we going?, Yasuda 987–990
Diabetes remission and relapse after metabolic surgery, Chen 1237–1238

education

Mental distress and health‐related quality of life among type 1 and type 2 diabetes patients using self‐monitoring of blood glucose: A cross‐sectional questionnaire study in Japan, Tanaka 1203–1211

elderly

Bullous pemphigoid associated with dipeptidyl peptidase‐4 inhibitors: A report of five cases, Yoshiji 445–447
Predicting the ability of elderly diabetes patients to acquire the insulin self‐injection technique based on the number of animal names recalled, Minami 623–628

elderly patients

Continuous glucose monitoring reveals hypoglycemia risk in elderly patients with type 2 diabetes mellitus, Ishikawa 69–74

endogenous glucose production

Comprehensive assessment of insulin resistance in non‐obese Asian Indian and Chinese men, Tan 1296–1303

endogenous insulin secretion capacity

Complete loss of insulin secretion capacity in type 1A diabetes patients during long‐term follow up, Uno 806–812

enteroendocrine cell

Distribution and hormonal characterization of primary murine L cells throughout the gastrointestinal tract, Suzuki 25–32

epidemiology

Trends in diabetic retinopathy and related medical practices among type 2 diabetes patients: Results from the National Insurance Service Survey 2006–2013, Song 173–178

error grid analysis

Performance of a new real‐time continuous glucose monitoring system: A multicenter pilot study, Zhou 286–293

estimated glomerular filtration rate

Renal function after intravitreal administration of vascular endothelial growth factor inhibitors in patients with diabetes and chronic kidney disease, Kameda 937–939

ethnic specificities

Assessing a new hip index as a risk predictor for diabetes mellitus, He 799–805

exercise

Physical activity and prevalence of erectile dysfunction in Japanese patients with type 2 diabetes mellitus: The Dogo Study, Minami 193–198

extensor digitorum longus

Effects of atorvastatin on autophagy in skeletal muscles of diabetic rats, Yang 753–761

fasting triglycerides and glucose

Clinical surrogate markers for predicting metabolic syndrome in middle‐aged and elderly Chinese, Li 411–418

fat‐free mass

Assessment of resting energy expenditure and body composition in Japanese pregnant women with diabetes, Eto 959–966

fat intake

Relationship between deterioration of glycated hemoglobin‐lowering effects in dipeptidyl peptidase‐4 inhibitor monotherapy and dietary habits: Retrospective analysis of Japanese individuals with type 2 diabetes, Kuwata 1153–1158

fatty liver

Impaired peripheral insulin sensitivity in non‐obese Japanese patients with type 2 diabetes mellitus and fatty liver, Furukawa 529–535

ferritin

Correlation between plasma ferritin level and gestational diabetes mellitus and its impact on fetal macrosomia, Wang 1354–1359

ficolin‐3

Ficolin‐3/adiponectin ratio for the prediction of gestational diabetes mellitus in pregnant women, Yuan 403–410

first phase of insulin secretion

Relationship between serum adipsin and the first phase of glucose‐stimulated insulin secretion in individuals with different glucose tolerance, Zhou 1128–1134

frailty

Low glycated hemoglobin level is associated with severity of frailty in Japanese elderly diabetes patients, Yanagita 419–425

fulminant type 1 diabetes

High risk of renal dysfunction in patients with fulminant type 1 diabetes, Takahashi 146–151
Insulin‐producing cells derived from ‘induced pluripotent stem cells’ of patients with fulminant type 1 diabetes: Vulnerability to cytokine insults and increased expression of apoptosis‐related genes, Hosokawa 481–493
Analysis of pancreatic volume in acute‐onset, slowly‐progressive and fulminant type 1 diabetes in a Japanese population, Sasamori 1091–1099

γ‐glutamyltransferase

Serum γ‐glutamyltransferase level and metabolic syndrome in children and adolescents: Korean National Health and Nutrition Examination Survey, Park 522–528

genetic engineering

Beta‐cell replacement strategies for diabetes, Kieffer 457–463

genomics

Precision medicine in diabetes prevention, classification and management, Xie 998–1015

gestational diabetes

One‐hour oral glucose tolerance test plasma glucose at gestational diabetes diagnosis is a common predictor of the need for insulin therapy in pregnancy and postpartum impaired glucose tolerance, Nishikawa 1370–1377

gestational diabetes mellitus

Preliminary screening of mutations in the glucokinase gene of Chinese patients with gestational diabetes, Wang 199–203
Gestational diabetes mellitus and first trimester pregnancy‐associated plasma protein A: A case–control study in a Chinese population, Xiao 204–210
Ficolin‐3/adiponectin ratio for the prediction of gestational diabetes mellitus in pregnant women, Yuan 403–410
Early second trimester maternal serum markers in the prediction of gestational diabetes mellitus, Zhao 967–974
Micro‐ribonucleic acid‐binding site variants of type 2 diabetes candidate loci predispose to gestational diabetes mellitus in Chinese Han women, Wang 1196–1202
Correlation between plasma ferritin level and gestational diabetes mellitus and its impact on fetal macrosomia, Wang 1354–1359
Lactation and progression to type 2 diabetes in patients with gestational diabetes mellitus: A systematic review and meta‐analysis of cohort studies, Feng 1360–1369

glimepiride

Glimepiride treatment in a patient with type A insulin resistance syndrome due to a novel heterozygous missense mutation in the insulin receptor gene, Huang 1075–1083

glucagon

Insulin deficiency with and without glucagon: A comparative study between total pancreatectomy and type 1 diabetes, Niwano 1084–1090
Sex differences in insulin and glucagon responses for glucose homeostasis in young healthy Japanese adults, Horie 1283–1287

glucagon‐like peptide 1

Weekly glucagon‐like peptide‐1 receptor agonist albiglutide as monotherapy improves glycemic parameters in Japanese patients with type 2 diabetes mellitus: A randomized, double‐blind, placebo‐controlled study, Nino 558–566

glucagon‐like peptide‐1 receptor agonist

Safety, tolerability and efficacy of lixisenatide as monotherapy in Japanese patients with type 2 diabetes mellitus: An open‐label, multicenter study, Seino 108–118
Safety, tolerability and efficacy of lixisenatide in combination with oral antidiabetic treatment in Japanese patients with type 2 diabetes: An open‐label, multicenter study, Seino 127‐136
Safety and efficacy of the combination of the glucagon‐like peptide‐1 receptor agonist liraglutide with an oral antidiabetic drug in Japanese patients with type 2 diabetes: Post‐hoc analysis of a randomized, 52‐week, open‐label, parallel‐group trial, Kiyosue 831–839

glucagon stimulation test

Normal meal tolerance test is preferable to the glucagon stimulation test in patients with type 2 diabetes that are not in a hyperglycemic state: Comparison with the change of C‐peptide immunoreactivity, Fujioka 274–278

glucokinase

Preliminary screening of mutations in the glucokinase gene of Chinese patients with gestational diabetes, Wang 199–203

glucose clamp(s)

Effects of a novel selective peroxisome proliferator‐activated receptor‐α modulator, pemafibrate, on hepatic and peripheral glucose uptake in patients with hypertriglyceridemia and insulin resistance, Matsuba 1323–1332

glucose intolerant

Relationship between urine pH and abnormal glucose tolerance in a community‐based study, Yoshida 769–775

glucose peak time

Delay in glucose peak time during the oral glucose tolerance test as an indicator of insulin resistance and insulin secretion in type 2 diabetes patients, Wang 1288–1295

glucose tolerance

One‐hour oral glucose tolerance test plasma glucose at gestational diabetes diagnosis is a common predictor of the need for insulin therapy in pregnancy and postpartum impaired glucose tolerance, Nishikawa 1370–1377

glutamine

Regulation of amino acid metabolism and α‐cell proliferation by glucagon, Hayashi 464–472

glycated hemoglobin

Twenty‐four‐hour variations in blood glucose level in Japanese type 2 diabetes patients based on continuous glucose monitoring, Hajime 75‐82
Low glycated hemoglobin level is associated with severity of frailty in Japanese elderly diabetes patients, Yanagita 419–425
Efficacy and safety of adding liraglutide to existing insulin regimens in Japanese patients with type 2 diabetes mellitus: A post‐hoc analysis of a phase 3 randomized clinical trial, Kaneko 840–849

glycemic control

Effectiveness and acceptability of continuous glucose monitoring for type 2 diabetes management: A narrative review, Taylor 713‐725
Impact of age at menarche on obesity and glycemic control in Japanese patients with type 2 diabetes: Fukuoka Diabetes Registry, Sumi 1216–1223

glycemic remission

Lower mean blood glucose during short‐term intensive insulin therapy is associated with long‐term glycemic remission in patients with newly diagnosed type 2 diabetes: Evidence‐based recommendations for standardization, Liu 908–916

glycemic variability

Comparison of the relationship between multiple parameters of glycemic variability and coronary plaque vulnerability assessed by virtual histology–intravascular ultrasound, Otowa‐Suematsu 610–615

gut microbiota

Understanding the role of the gut ecosystem in diabetes mellitus, Aw 5–12
Role of gut microbiota, bile acids and their cross‐talk in the effects of bariatric surgery on obesity and type 2 diabetes, Liu 13–20

hand function

Do we underestimate influences of diabetic mononeuropathy or polyneuropathy on hand functional performance and life quality?, Yang 179–185

hand neuropathy

Do we underestimate influences of diabetic mononeuropathy or polyneuropathy on hand functional performance and life quality?, Yang 179–185

health‐related quality of life

Mental distress and health‐related quality of life among type 1 and type 2 diabetes patients using self‐monitoring of blood glucose: A cross‐sectional questionnaire study in Japan, Tanaka 1203–1211

hedonic hunger

Association between hedonic hunger and glycemic control in non‐obese and obese patients with type 2 diabetes, Cheung 1135–1143

hemoperfusion

High‐volume continuous venovenous hemodiafiltration plus resin hemoperfusion improves severe metformin‐associated toxicity, Liu 975–978

Hepatocellular lipid content

Angiopoietin‐like protein 8/betatrophin correlates with hepatocellular lipid content independent of insulin resistance in non‐alcoholic fatty liver disease patients, Hong 952–958

high‐fat diet

Ipragliflozin improves mitochondrial abnormalities in renal tubules induced by a high‐fat diet, Takagi 1025–1032

human neonates

Immaturity of insulin secretion by pancreatic islets isolated from one human neonate, Henquin 270–273

hydrogen

Subcutaneous injection of hydrogen gas is a novel effective treatment for type 2 diabetes, Zhang 83–90

hypercholesterolemia

Dipeptidyl peptidase 4 inhibitor anagliptin ameliorates hypercholesterolemia in hypercholesterolemic mice through inhibition of intestinal cholesterol transport, Goto 1261–1269

hypertension

Switching from low‐dose thiazide diuretics to sodium–glucose cotransporter 2 inhibitor improves various metabolic parameters without affecting blood pressure in patients with type 2 diabetes and hypertension, Kimura 875–881

hypoglycemia

Continuous glucose monitoring reveals hypoglycemia risk in elderly patients with type 2 diabetes mellitus, Ishikawa 69–74
Characteristics of frequent emergency department users with type 2 diabetes mellitus in Korea, Ustulin 430–437
Incidence rate and patient characteristics of severe hypoglycemia in treated type 2 diabetes mellitus patients in Japan: Retrospective Diagnosis Procedure Combination database analysis, Ikeda 925–936

impaired glucose tolerance

Accelerated oligosaccharide absorption and altered serum metabolites during oral glucose tolerance test in young Japanese with impaired glucose tolerance, Miki 512–521

induced pluripotent stem cell

Insulin‐producing cells derived from ‘induced pluripotent stem cells’ of patients with fulminant type 1 diabetes: Vulnerability to cytokine insults and increased expression of apoptosis‐related genes, Hosokawa 481–493

induced pluripotent stem cells

iPSC technology‐based regenerative therapy for diabetes, Kondo 234–243

inflammation

Plasma interleukin‐22 levels are associated with prediabetes and type 2 diabetes in the Han Chinese population, Shen 33–38

Curcumin attenuates high glucose‐induced inflammatory injury through the reactive oxygen species–phosphoinositide 3‐kinase/protein kinase B–nuclear factor‐κB signaling pathway in rat thoracic aorta endothelial cells, Zhang 731–740

insulin

Insulin degludec in the first trimester of pregnancy: Report of two cases, Milluzzo 629–631
The current status of treatment‐related severe hypoglycemia in Japanese patients with diabetes mellitus: A report from the committee on a survey of severe hypoglycemia in the Japan Diabetes Society, Namba 642–656
Addition of dipeptidyl peptidase‐4 inhibitors to insulin treatment in type 2 diabetes patients: A meta‐analysis, Yang 813–821
Efficacy and safety of adding liraglutide to existing insulin regimens in Japanese patients with type 2 diabetes mellitus: A post‐hoc analysis of a phase 3 randomized clinical trial, Kaneko 840–849
Sodium–glucose cotransporter 2 inhibitor, tofogliflozin, shows better improvements of blood glucose and insulin secretion in patients with high insulin levels at baseline, Tobe 862–869
Sex differences in insulin and glucagon responses for glucose homeostasis in young healthy Japanese adults, Horie 1283–1287
Cross‐sectional survey of biosimilar insulin utilization in Asia: The Joint Asia Diabetes Evaluation Program, Gani 1312–1322
One‐hour oral glucose tolerance test plasma glucose at gestational diabetes diagnosis is a common predictor of the need for insulin therapy in pregnancy and postpartum impaired glucose tolerance, Nishikawa 1370–1377

insulin aspart‐30

Biphasic insulin aspart‐30 reduces glycemic variability to a greater degree than insulin detemir: A randomized controlled trial of once‐daily insulin regimens using continuous glucose monitoring, Tsujino 573–578

insulin degludec/insulin aspart

Efficacy and safety of switching from basal insulin to once‐daily insulin degludec/insulin aspart in Japanese patients with inadequately controlled type 2 diabetes: A 4‐week, randomized, open‐label, treat‐to‐target study, Nagai 567–572

insulin detemir

Biphasic insulin aspart‐30 reduces glycemic variability to a greater degree than insulin detemir: A randomized controlled trial of once‐daily insulin regimens using continuous glucose monitoring, Tsujino 573–578

insulin glargine

Open‐label, randomized study comparing basal insulin peglispro and insulin glargine, in combination with oral antihyperglycemic medications, in insulin‐naïve Asian patients with type 2 diabetes, Hirose 100–107

insulin‐like growth factor‐1

Pro‐apoptotic effects of micro‐ribonucleic acid‐365 on retinal neurons by targeting insulin‐like growth factor‐1 in diabetic rats: An *in vivo* and *in vitro* study, Zheng 1041–1051

insulin pump

Safety of the batteries and power units used in insulin pumps: A pilot cross‐sectional study by the Association for the Study of Innovative Diabetes Treatment in Japan, Murata 903–907

insulin requirement

Insulin deficiency with and without glucagon: A comparative study between total pancreatectomy and type 1 diabetes, Niwano 1084–1090

insulin resistance

Pancreatic fat content assessed by ^1^H magnetic resonance spectroscopy is correlated with insulin resistance, but not with insulin secretion, in Japanese individuals with normal glucose tolerance, Komada 505–511
Impaired peripheral insulin sensitivity in non‐obese Japanese patients with type 2 diabetes mellitus and fatty liver, Furukawa 529–535
Empagliflozin decreases remnant‐like particle cholesterol in type 2 diabetes patients with insulin resistance, Hattori 870–874
Treatment of a case of severe insulin resistance as a result of a *PIK3R1* mutation with a sodium–glucose cotransporter 2 inhibitor, Hamaguchi 1224–1227
Effects of a novel selective peroxisome proliferator‐activated receptor‐α modulator, pemafibrate, on hepatic and peripheral glucose uptake in patients with hypertriglyceridemia and insulin resistance, Matsuba 1323–1332

insulin secretion

Immaturity of insulin secretion by pancreatic islets isolated from one human neonate, Henquin 270–273
Pancreatic fat content assessed by ^1^H magnetic resonance spectroscopy is correlated with insulin resistance, but not with insulin secretion, in Japanese individuals with normal glucose tolerance, Komada 505–511
Serum adiponectin and insulin secretion: A direct or inverse association?, Nakamura 1106–1109

insulin self‐injection

Predicting the ability of elderly diabetes patients to acquire the insulin self‐injection technique based on the number of animal names recalled, Minami 623–628

insulin sensitivity

Delay in glucose peak time during the oral glucose tolerance test as an indicator of insulin resistance and insulin secretion in type 2 diabetes patients, Wang 1288–1295

insulin therapy

Real‐life glycemic control in patients with type 2 diabetes treated with insulin therapy: A prospective, longitudinal cohort study (Diabetes Distress and Care Registry at Tenri [DDCRT 9]), Fujita 294–302

insurance

Trends in diabetic retinopathy and related medical practices among type 2 diabetes patients: Results from the National Insurance Service Survey 2006–2013, Song 173–178

interleukin‐8

Clinical significance of serum interleukin‐8 and soluble tumor necrosis factor‐like weak inducer of apoptosis levels in patients with diabetic nephropathy, Liu 1182–1188

interleukin‐22

Plasma interleukin‐22 levels are associated with prediabetes and type 2 diabetes in the Han Chinese population, Shen 33–38

intestinal L cell

Distribution and hormonal characterization of primary murine L cells throughout the gastrointestinal tract, Suzuki 25–32

intravitreal injection

Renal function after intravitreal administration of vascular endothelial growth factor inhibitors in patients with diabetes and chronic kidney disease, Kameda 937–939

islet transplantation

Beta‐cell replacement strategies for diabetes, Kieffer 457–463

isolated islets

Immaturity of insulin secretion by pancreatic islets isolated from one human neonate, Henquin 270–273

Japan

Weekly glucagon‐like peptide‐1 receptor agonist albiglutide as monotherapy improves glycemic parameters in Japanese patients with type 2 diabetes mellitus: A randomized, double‐blind, placebo‐controlled study, Nino 558–566
Efficacy and safety of the G protein‐coupled receptor 119 agonist DS‐8500a in Japanese type 2 diabetes mellitus patients with inadequate glycemic control on sitagliptin: A phase 2 randomized placebo‐controlled study, Terauchi 1333–1341

Japanese

Fast‐acting insulin aspart in Japanese patients with type 1 diabetes: Faster onset, higher early exposure and greater early glucose‐lowering effect relative to insulin aspart, Shiramoto 303–310

Japanese patients

Safety, tolerability and efficacy of lixisenatide as monotherapy in Japanese patients with type 2 diabetes mellitus: An open‐label, multicenter study, Seino 108–118
Safety, tolerability and efficacy of lixisenatide in combination with oral antidiabetic treatment in Japanese patients with type 2 diabetes: An open‐label, multicenter study, Seino 127–136

Sodium‐glucose cotransporter‐2 inhibitor luseogliflozin added to glucagon‐like peptide 1 receptor agonist liraglutide improves glycemic control with bodyweight and fat mass reductions in Japanese patients with type 2 diabetes: A 52‐week, open‐label, single‐arm study, Seino 332–340

lactation

Lactation and progression to type 2 diabetes in patients with gestational diabetes mellitus: A systematic review and meta‐analysis of cohort studies, Feng 1360–1369

lanreotide

Marked alteration of glycemic profile surrounding lanreotide administration in acromegaly: A case report, Tanaka 223–225

leptin

Plasma soluble leptin receptor levels are associated with pancreatic β‐cell dysfunction in patients with type 2 diabetes, Morioka 55–62
Effects of pioglitazone treatment on blood leptin levels in patients with type 2 diabetes, Ida 917–924
Positive association of plasma leptin with sleep quality in obese type 2 diabetes patients, Hirota 1100–1105

Letter to the Editor

Aggravation of type 2 diabetes mellitus triggered by acute exacerbation of chronic thyroiditis (Hashimoto's disease), Takai 226–227
Intrinsic insulin secretion capacity might be preserved by discontinuing anti‐programmed cell death protein 1 antibody treatment in ‘anti‐programmed cell death protein 1 antibody‐induced’ fulminant type 1 diabetes, Sakai 448–449
Body mass index increase before 3 years‐of‐age augments the degree of insulin resistance corresponding to body mass index in adolescent girls, Arisaka 450–450
Short‐term changes in pancreatic α‐cell function after the onset of fulminant type 1 diabetes, Takahashi 636–637
Changes in heart rate in patients with type 2 diabetes mellitus after treatment with luseogliflozin: Subanalysis of placebo‐controlled, double‐blind clinical trials, Sano 638–641
Disease duration as an indicator of the efficacy of liraglutide in patients with type 2 diabetes mellitus, Wilbrink 979–980
Reply to the comment of Wilbrink *et al*. on Retrospective analysis of liraglutide and basal insulin combination therapy in Japanese type 2 diabetes: The association between remaining β‐cell function and the achievement of the HbA1c target 1 year after initiation, Usui 981–983
Administration of thiamazole for Graves’ disease might trigger the onset of type 1 diabetes, Mizutani 1228–1229
Status of glycemic control in elderly patients with cognitive impairment treated by general practitioners relative to the glycemic targets recommended for elderly patients by the Japan Diabetes Society/Japan Geriatrics Society Joint Committee: A retrospective analysis, Sugimoto 1230–1232
Onset of fulminant type 1 diabetes mellitus under immunotolerance status after long‐term therapy for chronic inflammatory demyelinating polyneuropathy, Shigemoto 1378–1380
Onset of type 1 diabetes mellitus and heparin‐induced thrombocytopenia in a patient with Basedow's disease and idiopathic thrombocytopenic purpura: Novel combination as autoimmune polyglandular syndrome, Kinoshita 1381–1382

lifestyle

Effectiveness and acceptability of continuous glucose monitoring for type 2 diabetes management: A narrative review, Taylor 713–725

linkage analysis

Genome‐wide scan for circulating vascular adhesion protein‐1 levels: MACROD2 as a potential transcriptional regulator of adipogenesis, Chang 1067–1074

lipid accumulation product

Clinical surrogate markers for predicting metabolic syndrome in middle‐aged and elderly Chinese, Li 411–418

lipid deposition

Effects of atorvastatin on autophagy in skeletal muscles of diabetic rats, Yang 753‐761

lipodystrophy

Case of lipoatrophic diabetes induced by juvenile dermatomyositis, Baba 632–635

lipohypertrophy

Prevalence of lipohypertrophy in insulin‐treated diabetes patients: A systematic review and meta‐analysis, Deng 536–543

lipopolysaccharide

Lipopolysaccharide inhibits hepatic gluconeogenesis in rats: The role of immune cells, Tanaka 494–504

liraglutide

Retrospective analysis of liraglutide and basal insulin combination therapy in Japanese type 2 diabetes patients: The association between remaining β‐cell function and the achievement of the glycated hemoglobin target 1 year after initiation, Usui 822–830
Safety and efficacy of the combination of the glucagon‐like peptide‐1 receptor agonist liraglutide with an oral antidiabetic drug in Japanese patients with type 2 diabetes: Post‐hoc analysis of a randomized, 52‐week, open‐label, parallel‐group trial, Kiyosue 831–839
Efficacy and safety of adding liraglutide to existing insulin regimens in Japanese patients with type 2 diabetes mellitus: A post‐hoc analysis of a phase 3 randomized clinical trial, Kaneko 840–849

liver

Lipopolysaccharide inhibits hepatic gluconeogenesis in rats: The role of immune cells, Tanaka 494–504

lixisenatide

Satisfaction of switching to combination therapy with lixisenatide and basal insulin in patients with type 2 diabetes receiving multiple daily insulin injection therapy: A randomized controlled trial, Miya 119–126
Safety, tolerability and efficacy of lixisenatide in combination with oral antidiabetic treatment in Japanese patients with type 2 diabetes: An open‐label, multicenter study, Seino 127–136

long‐acting insulin

Comparison of morning basal + 1 bolus insulin therapy (insulin glulisine + insulin glargine 300 U/mL vs insulin lispro + insulin glargine biosimilar) using continuous glucose monitoring: A randomized crossover study, Takeishi 91–99

long‐standing

Complete loss of insulin secretion capacity in type 1A diabetes patients during long‐term follow up, Uno 806–812

luseogliflozin

Sodium‐glucose cotransporter‐2 inhibitor luseogliflozin added to glucagon‐like peptide 1 receptor agonist liraglutide improves glycemic control with bodyweight and fat mass reductions in Japanese patients with type 2 diabetes: A 52‐week, open‐label, single‐arm study, Seino 332–340

MACRO domain containing 2 gene

Genome‐wide scan for circulating vascular adhesion protein‐1 levels: MACROD2 as a potential transcriptional regulator of adipogenesis, Chang 1067–1074

macrophages

Molecular mechanism of obesity‐induced ‘metabolic’ tissue remodeling, Tanaka 256–261

macrosomia

Correlation between plasma ferritin level and gestational diabetes mellitus and its impact on fetal macrosomia, Wang 1354–1359

maturity‐onset diabetes of the young

Maturity‐onset diabetes of the young as a model for elucidating the multifactorial origin of type 2 diabetes mellitus, Horikawa 704–712

meal tolerance test

Normal meal tolerance test is preferable to the glucagon stimulation test in patients with type 2 diabetes that are not in a hyperglycemic state: Comparison with the change of C‐peptide immunoreactivity, Fujioka 274–278

mean amplitude of glycemic excursions

Twenty‐four‐hour variations in blood glucose level in Japanese type 2 diabetes patients based on continuous glucose monitoring, Hajime 75–82

mean blood glucose

Lower mean blood glucose during short‐term intensive insulin therapy is associated with long‐term glycemic remission in patients with newly diagnosed type 2 diabetes: Evidence‐based recommendations for standardization, Liu 908–916

medication

Impact of obesity on annual medical expenditures and diabetes care in Japanese patients with type 2 diabetes mellitus, Kusunoki‐Tsuji 776–781

member B of the family with sequence similarity 3

Effects of circulating member B of the family with sequence similarity 3 on the risk of developing metabolic syndrome and its components: A 5‐year prospective study, Wang 782–788

menopause

Age at natural menopause and risk of diabetes in adult women: Findings from the China Kadoorie Biobank study in the Zhejiang area, Wang 762–768

meta‐analysis

Prevalence of lipohypertrophy in insulin‐treated diabetes patients: A systematic review and meta‐analysis, Deng 536–543
Comparison between sodium–glucose cotransporter 2 inhibitors and pioglitazone as additions to insulin therapy in type 2 diabetes patients: A systematic review with an indirect comparison meta‐analysis, Cho 882–892

metabolic syndrome

Prevalence of metabolic syndrome in Chinese psoriasis patients: A hospital‐based cross‐sectional study, Gui 39–43
Clinical surrogate markers for predicting metabolic syndrome in middle‐aged and elderly Chinese, Li 411–418
Serum γ‐glutamyltransferase level and metabolic syndrome in children and adolescents: Korean National Health and Nutrition Examination Survey, Park 522–528
Effects of circulating member B of the family with sequence similarity 3 on the risk of developing metabolic syndrome and its components: A 5‐year prospective study, Wang 782–788
Prevalence, treatment patterns and control rates of metabolic syndrome in a Chinese diabetic population: China Cardiometabolic Registries 3B study, Jing 789–798

metabolome

Accelerated oligosaccharide absorption and altered serum metabolites during oral glucose tolerance test in young Japanese with impaired glucose tolerance, Miki 512–521

metabolomics

Understanding the role of the gut ecosystem in diabetes mellitus, Aw 5–12
Metabolomics window into diabetic complications, Wu 244–255

metagenomics

Understanding the role of the gut ecosystem in diabetes mellitus, Aw 5‐12

mortality

impact of obesity on mortality in patients with diabetes: Meta‐analysis of 20 studies including 250,016 patients, Gao 44–54

metformin

Effects of dosage and dosing frequency on the efficacy and safety of high‐dose metformin in Japanese patients with type 2 diabetes mellitus, Kanto 587–593

metformin overdose

High‐volume continuous venovenous hemodiafiltration plus resin hemoperfusion improves severe metformin‐associated toxicity, Liu 975–978

metreleptin

Case of lipoatrophic diabetes induced by juvenile dermatomyositis, Baba 632–635

microalbuminuria

High risk of renal dysfunction in patients with fulminant type 1 diabetes, Takahashi 146–151

microribonucleic acid‐192

Microribonucleic acid‐192 as a specific biomarker for the early diagnosis of diabetic kidney disease, Yang 602–609

micro‐ribonucleic acid‐365

Pro‐apoptotic effects of micro‐ribonucleic acid‐365 on retinal neurons by targeting insulin‐like growth factor‐1 in diabetic rats: An *in vivo* and *in vitro* study, Zheng 1041–1051

microvascular complications

Severity and multiplicity of microvascular complications are associated with QT interval prolongation in patients with type 2 diabetes, Kobayashi 946–951

mitochondria

Ipragliflozin improves mitochondrial abnormalities in renal tubules induced by a high‐fat diet, Takagi 1025–1032

monoclonal antibodies

Antibodies against H1N1 influenza virus cross‐react with α‐cells of pancreatic islets, Qi 265–269

monotherapy failure

Failure of monotherapy in clinical practice in patients with type 2 diabetes: The Korean National Diabetes Program, Jeon 1144–1152

muscle strength

Diabetic polyneuropathy is a risk factor for decline of lower extremity strength in patients with type 2 diabetes, Nomura 186–192
Regular exercise behavior is related to lower extremity muscle strength in patients with type 2 diabetes: Data from the Multicenter Survey of the Isometric Lower Extremity Strength in Type 2 Diabetes study, Nomura 426‐429

mutation

Treatment of a case of severe insulin resistance as a result of a *PIK3R1* mutation with a sodium–glucose cotransporter 2 inhibitor, Hamaguchi 1224–1227

neurodegeneration

Diabetic neuropathy and the sensory neuron: New aspects of pathogenesis and their treatment implications, Kobayashi 1239–1254

nivolumab

Aggravation of diabetes, and incompletely deficient insulin secretion in a case with type 1 diabetes‐resistant human leukocyte antigen DRB1*15:02 treated with nivolumab, Matsumura 438–441

nocturia

Dietary intake habits and the prevalence of nocturia in Japanese patients with type 2 diabetes mellitus, Furukawa 279–285

non‐alcoholic fatty liver disease

Angiopoietin‐like protein 8/betatrophin correlates with hepatocellular lipid content independent of insulin resistance in non‐alcoholic fatty liver disease patients, Hong 952–958

non‐high‐density lipoprotein cholesterol

Non‐high‐density lipoprotein cholesterol is more informative than traditional cholesterol indices in predicting diabetes risk for women with normal glucose tolerance, Liu 1304–1311

non‐obese

Impaired peripheral insulin sensitivity in non‐obese Japanese patients with type 2 diabetes mellitus and fatty liver, Furukawa 529–535

non‐obese Asian Indian

Comprehensive assessment of insulin resistance in non‐obese Asian Indian and Chinese men, Tan 1296–1303

novel mutation

Glimepiride treatment in a patient with type A insulin resistance syndrome due to a novel heterozygous missense mutation in the insulin receptor gene, Huang 1075–1083

obesity

Molecular mechanism of obesity‐induced ‘metabolic’ tissue remodeling, Tanaka 256–261
Impact of obesity on annual medical expenditures and diabetes care in Japanese patients with type 2 diabetes mellitus, Kusunoki‐Tsuji 776–781
Positive association of plasma leptin with sleep quality in obese type 2 diabetes patients, Hirota 1100–1105
Association between hedonic hunger and glycemic control in non‐obese and obese patients with type 2 diabetes, Cheung 1135–1143

obstructive sleep apnea syndrome

Application of 3C insulin pump system in combination with non‐invasive ventilation in the treatment of a patient with type 2 diabetes and obstructive sleep apnea syndrome, Dong 442–444

once‐weekly dipeptidyl peptidase‐4 inhibitor

Efficacy and safety of once‐weekly oral trelagliptin switched from once‐daily dipeptidyl peptidase‐4 inhibitor in patients with type 2 diabetes mellitus: An open‐label, phase 3 exploratory study, Inagaki 354–359

oral glucose tolerance test

Accelerated oligosaccharide absorption and altered serum metabolites during oral glucose tolerance test in young Japanese with impaired glucose tolerance, Miki 512–521

oral hypoglycemic agents

Dipeptidyl peptidase‐4 inhibitors as preferable oral hypoglycemic agents in terms of treatment satisfaction: Results from a multicenter, 12‐week, open label, randomized controlled study in Japan (PREFERENCE 4 study), Ishii 137–145

orthostatic hypotension

Blood pressure circadian rhythms and adverse outcomes in type 2 diabetes patients diagnosed with orthostatic hypotension, Chang 383–388

pancreatic β‐cell function

Delay in glucose peak time during the oral glucose tolerance test as an indicator of insulin resistance and insulin secretion in type 2 diabetes patients, Wang 1288–1295

pancreatic fat

Pancreatic fat content assessed by ^1^H magnetic resonance spectroscopy is correlated with insulin resistance, but not with insulin secretion, in Japanese individuals with normal glucose tolerance, Komada 505–511

pancreatic volume

Analysis of pancreatic volume in acute‐onset, slowly‐progressive and fulminant type 1 diabetes in a Japanese population, Sasamori 1091–1099

penetrance

Maturity‐onset diabetes of the young as a model for elucidating the multifactorial origin of type 2 diabetes mellitus, Horikawa 704–712

peripheral neuropathy

Determination of peripheral neuropathy in high‐fat diet fed low‐dose streptozotocin‐treated female C57Bl/6J mice and Sprague–Dawley rats, Coppey 1033–1040

pH

Relationship between urine pH and abnormal glucose tolerance in a community‐based study, Yoshida 769–775

pharmacodynamics

Fast‐acting insulin aspart in Japanese patients with type 1 diabetes: Faster onset, higher early exposure and greater early glucose‐lowering effect relative to insulin aspart, Shiramoto 303–310

pharmacogenetics

Precision medicine in diabetes prevention, classification and management, Xie 998–1015

pharmacokinetics

Fast‐acting insulin aspart in Japanese patients with type 1 diabetes: Faster onset, higher early exposure and greater early glucose‐lowering effect relative to insulin aspart, Shiramoto 303–310

physical activity

Physical activity and prevalence of erectile dysfunction in Japanese patients with type 2 diabetes mellitus: The Dogo Study, Minami 193–198

*PIK3R1*

Treatment of a case of severe insulin resistance as a result of a *PIK3R1* mutation with a sodium–glucose cotransporter 2 inhibitor, Hamaguchi 1224–1227

pioglitazone

Case of lipoatrophic diabetes induced by juvenile dermatomyositis, Baba 632–635
Comparison between sodium–glucose cotransporter 2 inhibitors and pioglitazone as additions to insulin therapy in type 2 diabetes patients: A systematic review with an indirect comparison meta‐analysis, Cho 882–892

postoperative bleeding

Predictors of postoperative bleeding after vitrectomy for vitreous hemorrhage in patients with diabetic retinopathy, Motoda 940–945

postprandial hyperglycemia

Postprandial glucose‐lowering effect of premeal consumption of protein‐enriched, dietary fiber‐fortified bar in individuals with type 2 diabetes mellitus or normal glucose tolerance, Bae 1110–1118

precision medicine

Precision medicine in diabetes prevention, classification and management, Xie 998–1015

prediabetes

Prediabetes awareness among Southeastern European physicians, Kokic 544–548

prediction

Early second trimester maternal serum markers in the prediction of gestational diabetes mellitus, Zhao 967–974

predictive value

Gestational diabetes mellitus and first trimester pregnancy‐associated plasma protein A: A case–control study in a Chinese population, Xiao 204–210

pregnancy‐associated plasma protein A

Gestational diabetes mellitus and first trimester pregnancy‐associated plasma protein A: A case–control study in a Chinese population, Xiao 204–210

prevalence

Prevalence of metabolic syndrome in Chinese psoriasis patients: A hospital‐based cross‐sectional study, Gui 39–43

primary prevention

Prediabetes awareness among Southeastern European physicians, Kokic 544–548
Relationship between social engagement and diabetes incidence in a middle‐aged population: Results from a longitudinal nationwide survey in Japan, Shibayama 1060–1066
Non‐high‐density lipoprotein cholesterol is more informative than traditional cholesterol indices in predicting diabetes risk for women with normal glucose tolerance, Liu 1304–1311

prognostic marker

Tryptophan as a surrogate prognostic marker for diabetic nephropathy, Chou 366–374

prospective study

Effects of circulating member B of the family with sequence similarity 3 on the risk of developing metabolic syndrome and its components: A 5‐year prospective study, Wang 782–788

psoriasis

Prevalence of metabolic syndrome in Chinese psoriasis patients: A hospital‐based cross‐sectional study, Gui 39–43

psychological insulin resistance

Translation and validation of the Insulin Treatment Appraisal Scale in Hong Kong primary care patients, Lee 311–320

psychometric validation

Translation and validation of the Insulin Treatment Appraisal Scale in Hong Kong primary care patients, Lee 311–320

psychosocial factors

Association between poor psychosocial conditions and diabetic nephropathy in Japanese type 2 diabetes patients: A cross‐sectional study, Ninomiya 162–172

pump

Application of 3C insulin pump system in combination with non‐invasive ventilation in the treatment of a patient with type 2 diabetes and obstructive sleep apnea syndrome, Dong 442–444

pulmonary vascular remodeling

Cell death‐inducing DFF45‐like effector C gene silencing alleviates pulmonary vascular remodeling in a type 2 diabetic rat model, Sui 741–752

QT interval

Severity and multiplicity of microvascular complications are associated with QT interval prolongation in patients with type 2 diabetes, Kobayashi 946–951

quality of life

Do we underestimate influences of diabetic mononeuropathy or polyneuropathy on hand functional performance and life quality?, Yang 179–185

randomized controlled study

Dipeptidyl peptidase‐4 inhibitors as preferable oral hypoglycemic agents in terms of treatment satisfaction: Results from a multicenter, 12‐week, open label, randomized controlled study in Japan (PREFERENCE 4 study), Ishii 137–145

randomized controlled trial

Efficacy and safety of the G protein‐coupled receptor 119 agonist DS‐8500a in Japanese type 2 diabetes mellitus patients with inadequate glycemic control on sitagliptin: A phase 2 randomized placebo‐controlled study, Terauchi 1333–1341

randomized trial

Efficacy and safety of switching from basal insulin to once‐daily insulin degludec/insulin aspart in Japanese patients with inadequately controlled type 2 diabetes: A 4‐week, randomized, open‐label, treat‐to‐target study, Nagai 567–572

rat thoracic aorta endothelial cells

Curcumin attenuates high glucose‐induced inflammatory injury through the reactive oxygen species–phosphoinositide 3‐kinase/protein kinase B–nuclear factor‐κB signaling pathway in rat thoracic aorta endothelial cells, Zhang 731–740

regular exercise

Regular exercise behavior is related to lower extremity muscle strength in patients with type 2 diabetes: Data from the Multicenter Survey of the Isometric Lower Extremity Strength in Type 2 Diabetes study, Nomura 426–429

reimbursement

Factors associated with greater benefit of a national reimbursement policy for blood glucose test strips in adult patients with type 1 diabetes: A prospective cohort study, Jin 549–557

remission of hyperglycemia

Remission of hyperglycemia after withdrawal of oral antidiabetic drugs in Japanese patients with early‐stage type 2 diabetes, Hirukawa 1119–1127

remnant lipoprotein

Empagliflozin decreases remnant‐like particle cholesterol in type 2 diabetes patients with insulin resistance, Hattori 870–874

renal dysfunction

High risk of renal dysfunction in patients with fulminant type 1 diabetes, Takahashi 146–151

resting energy expenditure

Assessment of resting energy expenditure and body composition in Japanese pregnant women with diabetes, Eto 959–966

risk model

Development and validation of risk models to predict the 7‐year risk of type 2 diabetes: The Japan Epidemiology Collaboration on Occupational Health Study, Hu 1052–1059

screening

Prevention of renal failure in Chinese patients with newly diagnosed type 2 diabetes: A cost‐effectiveness analysis, Wu 152–161

seasonal fluctuation in hemoglobin A1c

Analysis of the effect of seasonal administration on the efficacy of sitagliptin: Subanalysis of the Januvia Multicenter Prospective Trial in Type 2 Diabetes Study, Sakura 1159–1166

selective peroxisome proliferator‐activated receptor‐α modulator

Effects of a novel selective peroxisome proliferator‐activated receptor‐α modulator, pemafibrate, on hepatic and peripheral glucose uptake in patients with hypertriglyceridemia and insulin resistance, Matsuba 1323–1332

self‐monitoring of blood glucose

Mental distress and health‐related quality of life among type 1 and type 2 diabetes patients using self‐monitoring of blood glucose: A cross‐sectional questionnaire study in Japan, Tanaka 1203–1211

sensory ganglia

Diabetic neuropathy and the sensory neuron: New aspects of pathogenesis and their treatment implications, Kobayashi, 1239–1254

serum marker

Early second trimester maternal serum markers in the prediction of gestational diabetes mellitus, Zhao 967–974

severe hypoglycemia

The current status of treatment‐related severe hypoglycemia in Japanese patients with diabetes mellitus: A report from the committee on a survey of severe hypoglycemia in the Japan Diabetes Society, Namba 642–656

Sex

Sex differences in insulin and glucagon responses for glucose homeostasis in young healthy Japanese adults, Horie 1283–1287

sexual function

Physical activity and prevalence of erectile dysfunction in Japanese patients with type 2 diabetes mellitus: The Dogo Study, Minami 193–198

short‐term intensive insulin therapy

Lower mean blood glucose during short‐term intensive insulin therapy is associated with long‐term glycemic remission in patients with newly diagnosed type 2 diabetes: Evidence‐based recommendations for standardization, Liu 908–916

single‐nucleotide polymorphism

Association between 1p13 polymorphisms and peripheral arterial disease in a Chinese population with diabetes, Qin 1189–1195

sitagliptin

Analysis of the effect of seasonal administration on the efficacy of sitagliptin: Subanalysis of the Januvia Multicenter Prospective Trial in Type 2 Diabetes Study, Sakura 1159–1166

skeletal muscle insulin sensitivity

Comprehensive assessment of insulin resistance in non‐obese Asian Indian and Chinese men, Tan 1296–1303

sleep

Characteristics of sleep–wake cycle and sleep duration in Japanese type 2 diabetes patients with visceral fat accumulation, Fukuda 63–68

sleep apnea

Sleep apnea and type 2 diabetes, Muraki 991–997

sleep quality

Positive association of plasma leptin with sleep quality in obese type 2 diabetes patients, Hirota 1100‐1105

social determinants of health

Relationship between social engagement and diabetes incidence in a middle‐aged population: Results from a longitudinal nationwide survey in Japan, Shibayama 1060–1066

social psychology

Cross‐cultural comparison of predictors for self‐care behaviors in patients with type 2 diabetes, Ikeda 1212–1215

social support

Association between poor psychosocial conditions and diabetic nephropathy in Japanese type 2 diabetes patients: A cross‐sectional study, Ninomiya 162–172

sodium–glucose cotransporter 2

Ipragliflozin improves mitochondrial abnormalities in renal tubules induced by a high‐fat diet, Takagi 1025–1032

sodium–glucose cotransporter 2 inhibitor

Efficacy of ipragliflozin as monotherapy or as add‐on therapy with other oral antidiabetic medications for treating type 2 diabetes in Japanese patients with inadequate glycemic control: A subgroup analysis based on patient characteristics, Osonoi 341–353
Empagliflozin decreases remnant‐like particle cholesterol in type 2 diabetes patients with insulin resistance, Hattori 870–874
Switching from low‐dose thiazide diuretics to sodium–glucose cotransporter 2 inhibitor improves various metabolic parameters without affecting blood pressure in patients with type 2 diabetes and hypertension, Kimura 875–881

sodium–glucose cotransporter 2 inhibitors

No disparity of the efficacy and all‐cause mortality between Asian and non‐Asian type 2 diabetes patients with sodium–glucose cotransporter 2 inhibitors treatment: A meta‐analysis, Cai 850–861
Comparison between sodium–glucose cotransporter 2 inhibitors and pioglitazone as additions to insulin therapy in type 2 diabetes patients: A systematic review with an indirect comparison meta‐analysis, Cho 882–892

soluble leptin receptor

Plasma soluble leptin receptor levels are associated with pancreatic β‐cell dysfunction in patients with type 2 diabetes, Morioka 55–62

soluble tumor necrosis factor‐like weak inducer of apoptosis

Clinical significance of serum interleukin‐8 and soluble tumor necrosis factor‐like weak inducer of apoptosis levels in patients with diabetic nephropathy, Liu 1182–1188

spleen

Lipopolysaccharide inhibits hepatic gluconeogenesis in rats: The role of immune cells, Tanaka 494–504

stem cell

Beta‐cell replacement strategies for diabetes, Kieffer 457–463

subcutaneous administration

Subcutaneous injection of hydrogen gas is a novel effective treatment for type 2 diabetes, Zhang 83–90

sulfonylureas

The current status of treatment‐related severe hypoglycemia in Japanese patients with diabetes mellitus: A report from the committee on a survey of severe hypoglycemia in the Japan Diabetes Society, Namba 642–656

technology

Effectiveness and acceptability of continuous glucose monitoring for type 2 diabetes management: A narrative review, Taylor 713–725

thiazide diuretics

Switching from low‐dose thiazide diuretics to sodium–glucose cotransporter 2 inhibitor improves various metabolic parameters without affecting blood pressure in patients with type 2 diabetes and hypertension, Kimura 875–881

thiazolidinediones

Effects of pioglitazone treatment on blood leptin levels in patients with type 2 diabetes, Ida 917–924

threshold

Grading effect of abnormal glucose status on arterial stiffness and a new threshold of 2‐h post‐load glucose based on a Chinese community study, Liu 616–622

tissue microarrays

Antibodies against H1N1 influenza virus cross‐react with α‐cells of pancreatic islets, Qi 265–269

tofogliflozin

Sodium–glucose cotransporter 2 inhibitor, tofogliflozin, shows better improvements of blood glucose and insulin secretion in patients with high insulin levels at baseline, Tobe 862–869

total pancreatectomy

Insulin deficiency with and without glucagon: A comparative study between total pancreatectomy and type 1 diabetes, Niwano 1084–1090

transcultural translation

Translation and validation of the Insulin Treatment Appraisal Scale in Hong Kong primary care patients, Lee 311–320

treatment satisfaction

Satisfaction of switching to combination therapy with lixisenatide and basal insulin in patients with type 2 diabetes receiving multiple daily insulin injection therapy: A randomized controlled trial, Miya 119–126
Dipeptidyl peptidase‐4 inhibitors as preferable oral hypoglycemic agents in terms of treatment satisfaction: Results from a multicenter, 12‐week, open label, randomized controlled study in Japan (PREFERENCE 4 study), Ishii 137–145

trelagliptin

Efficacy and safety of once‐weekly oral trelagliptin switched from once‐daily dipeptidyl peptidase‐4 inhibitor in patients with type 2 diabetes mellitus: An open‐label, phase 3 exploratory study, Inagaki 354–359

tryptophan

Tryptophan as a surrogate prognostic marker for diabetic nephropathy, Chou 366–374

type 1 diabetes

Aggravation of diabetes, and incompletely deficient insulin secretion in a case with type 1 diabetes‐resistant human leukocyte antigen DRB1*15:02 treated with nivolumab, Matsumura 438–441
Analysis of pancreatic volume in acute‐onset, slowly‐progressive and fulminant type 1 diabetes in a Japanese population, Sasamori 1091–1099
Glycemic extremes are related to cognitive dysfunction in children with type 1 diabetes: A meta‐analysis, He 1342–1353

type 1 diabetes mellitus

Factors associated with greater benefit of a national reimbursement policy for blood glucose test strips in adult patients with type 1 diabetes: A prospective cohort study, Jin 549–557

type 2 diabetes

Plasma interleukin‐22 levels are associated with prediabetes and type 2 diabetes in the Han Chinese population, Shen 33–38
Open‐label, randomized study comparing basal insulin peglispro and insulin glargine, in combination with oral antihyperglycemic medications, in insulin‐naïve Asian patients with type 2 diabetes, Hirose 100–107
Satisfaction of switching to combination therapy with lixisenatide and basal insulin in patients with type 2 diabetes receiving multiple daily insulin injection therapy: A randomized controlled trial, Miya 119–126
Diabetic polyneuropathy is a risk factor for decline of lower extremity strength in patients with type 2 diabetes, Nomura 186–192
Real‐life glycemic control in patients with type 2 diabetes treated with insulin therapy: A prospective, longitudinal cohort study (Diabetes Distress and Care Registry at Tenri [DDCRT 9]), Fujita 294–302
Characteristics of carotid atherosclerosis in elderly patients with type 2 diabetes at different disease course, and the intervention by statins in very elderly patients, Chen 389–395
Ratio of visceral‐to‐subcutaneous fat area predicts cardiovascular events in patients with type 2 diabetes, Fukuda 396–402
Low glycated hemoglobin level is associated with severity of frailty in Japanese elderly diabetes patients, Yanagita 419–425
Regular exercise behavior is related to lower extremity muscle strength in patients with type 2 diabetes: Data from the Multicenter Survey of the Isometric Lower Extremity Strength in Type 2 Diabetes study, Nomura 426–429
Prediabetes awareness among Southeastern European physicians, Kokic 544–548
Effects of dosage and dosing frequency on the efficacy and safety of high‐dose metformin in Japanese patients with type 2 diabetes mellitus, Kanto, 587–593
Current status of achieving blood pressure target and its clinical correlates in Japanese type 2 diabetes (JDDM45), Yokoyama 594–601
Addition of dipeptidyl peptidase‐4 inhibitors to insulin treatment in type 2 diabetes patients: A meta‐analysis, Yang 813–821
Safety and efficacy of the combination of the glucagon‐like peptide‐1 receptor agonist liraglutide with an oral antidiabetic drug in Japanese patients with type 2 diabetes: Post‐hoc analysis of a randomized, 52‐week, open‐label, parallel‐group trial, Kiyosue 831–839
No disparity of the efficacy and all‐cause mortality between Asian and non‐Asian type 2 diabetes patients with sodium–glucose cotransporter 2 inhibitors treatment: A meta‐analysis, Cai 850–861
Efficacy and safety of combination therapy with an α‐glucosidase inhibitor and a dipeptidyl peptidase‐4 inhibitor in patients with type 2 diabetes mellitus: A systematic review with meta‐analysis, Min 893–902
Incidence rate and patient characteristics of severe hypoglycemia in treated type 2 diabetes mellitus patients in Japan: Retrospective Diagnosis Procedure Combination database analysis, Ikeda 925–936
Determination of peripheral neuropathy in high‐fat diet fed low‐dose streptozotocin‐treated female C57Bl/6J mice and Sprague–Dawley rats, Coppey 1033–1040
Development and validation of risk models to predict the 7‐year risk of type 2 diabetes: The Japan Epidemiology Collaboration on Occupational Health Study, Hu 1052–1059
Remission of hyperglycemia after withdrawal of oral antidiabetic drugs in Japanese patients with early‐stage type 2 diabetes, Hirukawa 1119–1127
Association between hedonic hunger and glycemic control in non‐obese and obese patients with type 2 diabetes, Cheung 1135–1143
Failure of monotherapy in clinical practice in patients with type 2 diabetes: The Korean National Diabetes Program, Jeon 1144–1152
Micro‐ribonucleic acid‐binding site variants of type 2 diabetes candidate loci predispose to gestational diabetes mellitus in Chinese Han women, Wang 1196–1202
Non‐high‐density lipoprotein cholesterol is more informative than traditional cholesterol indices in predicting diabetes risk for women with normal glucose tolerance, Liu 1304–1311

type 2 diabetes mellitus

Characteristics of sleep–wake cycle and sleep duration in Japanese type 2 diabetes patients with visceral fat accumulation, Fukuda 63–68
Meta‐analysis and critical review on the efficacy and safety of alpha‐glucosidase inhibitors in Asian and non‐Asian populations, Gao 321–331
Efficacy of ipragliflozin as monotherapy or as add‐on therapy with other oral antidiabetic medications for treating type 2 diabetes in Japanese patients with inadequate glycemic control: A subgroup analysis based on patient characteristics, Osonoi 341–353
Efficacy and safety of once‐weekly oral trelagliptin switched from once‐daily dipeptidyl peptidase‐4 inhibitor in patients with type 2 diabetes mellitus: An open‐label, phase 3 exploratory study, Inagaki 354–359
Comparison of effects of anagliptin and alogliptin on serum lipid profile in type 2 diabetes mellitus patients, Kurozumi 360–365
Weekly glucagon‐like peptide‐1 receptor agonist albiglutide as monotherapy improves glycemic parameters in Japanese patients with type 2 diabetes mellitus: A randomized, double‐blind, placebo‐controlled study, Nino 558–566
Maturity‐onset diabetes of the young as a model for elucidating the multifactorial origin of type 2 diabetes mellitus, Horikawa 704–712
Effects of pioglitazone treatment on blood leptin levels in patients with type 2 diabetes, Ida 917–924
Severity and multiplicity of microvascular complications are associated with QT interval prolongation in patients with type 2 diabetes, Kobayashi 946–951
Relationship between serum adipsin and the first phase of glucose‐stimulated insulin secretion in individuals with different glucose tolerance, Zhou 1128–1134
Analysis of the effect of seasonal administration on the efficacy of sitagliptin: Subanalysis of the Januvia Multicenter Prospective Trial in Type 2 Diabetes Study, Sakura 1159–1166
Impact of age at menarche on obesity and glycemic control in Japanese patients with type 2 diabetes: Fukuoka Diabetes Registry, Sumi 1216–1223
Efficacy and safety of the G protein‐coupled receptor 119 agonist DS‐8500a in Japanese type 2 diabetes mellitus patients with inadequate glycemic control on sitagliptin: A phase 2 randomized placebo‐controlled study, Terauchi 1333–1341
Lactation and progression to type 2 diabetes in patients with gestational diabetes mellitus: A systematic review and meta‐analysis of cohort studies, Feng 1360–1369

Type A insulin resistance syndrome

Glimepiride treatment in a patient with type A insulin resistance syndrome due to a novel heterozygous missense mutation in the insulin receptor gene, Huang 1075–1083

ultra‐rapid‐acting insulin

Comparison of morning basal + 1 bolus insulin therapy (insulin glulisine + insulin glargine 300 U/mL vs insulin lispro + insulin glargine biosimilar) using continuous glucose monitoring: A randomized crossover study, Takeishi 91–99

urine

Relationship between urine pH and abnormal glucose tolerance in a community‐based study, Yoshida 769–775

variants

Micro‐ribonucleic acid‐binding site variants of type 2 diabetes candidate loci predispose to gestational diabetes mellitus in Chinese Han women, Wang 1196–1202

vascular adhesion protein‐1

Genome‐wide scan for circulating vascular adhesion protein‐1 levels: MACROD2 as a potential transcriptional regulator of adipogenesis, Chang 1067–1074

vascular endothelial growth factor

Renal function after intravitreal administration of vascular endothelial growth factor inhibitors in patients with diabetes and chronic kidney disease, Kameda 937–939

vegetable

Dietary intake habits and the prevalence of nocturia in Japanese patients with type 2 diabetes mellitus, Furukawa 279–285

ventilation

Application of 3C insulin pump system in combination with non‐invasive ventilation in the treatment of a patient with type 2 diabetes and obstructive sleep apnea syndrome, Dong 442–444

verbal fluency tests

Predicting the ability of elderly diabetes patients to acquire the insulin self‐injection technique based on the number of animal names recalled, Minami 623–628

very elderly patients with diabetes

Characteristics of carotid atherosclerosis in elderly patients with type 2 diabetes at different disease course, and the intervention by statins in very elderly patients, Chen 389–395

virtual histology–intravascular ultrasound

Comparison of the relationship between multiple parameters of glycemic variability and coronary plaque vulnerability assessed by virtual histology–intravascular ultrasound, Otowa‐Suematsu 610–615

visceral fat

Characteristics of sleep–wake cycle and sleep duration in Japanese type 2 diabetes patients with visceral fat accumulation, Fukuda 63–68

visceral‐to‐subcutaneous fat ratio

Ratio of visceral‐to‐subcutaneous fat area predicts cardiovascular events in patients with type 2 diabetes, Fukuda 396–402

vitamin D

Role of vitamin D in energy and bone metabolism in postmenopausal women with type 2 diabetes mellitus: A 6‐month follow‐up evaluation, Ogata 211–222
Association between serum 25‐hydroxyvitamin D and glycated hemoglobin levels in type 2 diabetes patients with chronic kidney disease, Lim 375–382

vitrectomy

Predictors of postoperative bleeding after vitrectomy for vitreous hemorrhage in patients with diabetic retinopathy, Motoda 940–945

whey proteins

Postprandial glucose‐lowering effect of premeal consumption of protein‐enriched, dietary fiber‐fortified bar in individuals with type 2 diabetes mellitus or normal glucose tolerance, Bae 1110–1118

withdrawal of oral antidiabetic drugs

Remission of hyperglycemia after withdrawal of oral antidiabetic drugs in Japanese patients with early‐stage type 2 diabetes, Hirukawa 1119–1127

